# A 400-Gb/s WDM-PAM4 OWC system through the free-space transmission with a water–air–water link

**DOI:** 10.1038/s41598-021-01006-x

**Published:** 2021-11-02

**Authors:** Hai-Han Lu, Chung-Yi Li, Xu-Hong Huang, Poh-Suan Chang, Yu-Ting Chen, Yan-Yu Lin, Chen-Xuan Liu, Ting Ko

**Affiliations:** 1 Institute of Electro-Optical Engineering, National Taipei University of Technology, Taipei, 10608 Taiwan; 2 Department of Communication Engineering, National Taipei University, New Taipei City, 23741 Taiwan; 3 The School of Information Science and Engineering, Fujian University of Technology, Fujian, 350118 China

**Keywords:** Engineering, Optics and photonics

## Abstract

A 400-Gb/s wavelength-division-multiplexing (WDM) four-level pulse amplitude modulation (PAM4) optical wireless communication (OWC) system through a 200-m free-space transmission with either an 8.8-m piped water–air–piped water link or a 6.5-m turbid water–air–turbid water link is successfully constructed. Incorporating PAM4 modulation with an 8-wavelength WDM scheme greatly increases the total transmission rate of the WDM-PAM4 OWC system to 400 Gb/s (50 Gb/s/λ × 8 λs). By adopting doublet lenses in free-space transmission, a laser beam reducer/expander and a reflective spatial light modulator (SLM) with an angle expander through the water–air–water link, good bit error rate performance and acceptable PAM4 eye diagrams are obtained. Using a reflective SLM with an angle expander not only adaptively adjusts the laser beam, but also effectively solves the oceanic engineering problems. This demonstrates WDM-PAM4 OWC system outperforms existing OWC systems through the free-space transmission with an air–water–air link because it can solve the practical engineering problems in actual oceanic environments.

## Introduction

Optical wireless communication (OWC) is a form of optical communication using laser beam or light-emitting diode light to deliver optical signals in an unguided environment. Free-space optical (FSO) communication is a type of OWC that transmits optical signals via a laser beam in free-space. FSO has received extensive attention in research on the problem of high-speed free-space transmissions^1–5^. Underwater wireless laser transmission (UWLT) system is another type of OWC that transmits optical signals via a laser beam through an underwater link. UWLT has attracted widespread attention in research on the problem of high-speed underwater links^6–11^. Given the characteristics of FSO communication and UWLT system, they can provide high transmission rate over a free-space transmission with an underwater link. With the rapid progress of FSO communication and UWLT system, constructing a high-speed FSO-UWLT convergence is vitally important^12,13^. In an actual scenario, no UWLT system passes through the air–water–air interface as the laser beam should flee or enter from the top of the water^14,15^. Sending a laser beam through the air–water–air interface cannot solve the problem currently being encountered in ocean engineering. A UWLT system passes through the water–air–water interface is more practical and can resolve the problem that the oceanic engineering is currently encountering. Our previous research showed a 50-Gb/s four-level pulse amplitude modulation (PAM4) UWLT system passing through a turbid water–air–turbid water link^16^. However, it is just a UWLT system passing through the turbid water–air–turbid water link. It cannot satisfy the demand to incorporate the free-space transmission with a water–air–water link. An OWC system through the free-space transmission with a water–air–water link is thus built to meet the high transmission rate requirement and resolve the practical engineering problem. In this research, a 400-Gb/s wavelength-division-multiplexing (WDM) PAM4 OWC system passing through a 200-m free-space transmission with either an 8.8-m piped water–air–piped water link or a 6.5-m turbid water–air–turbid water link is proposed and constructed. It shows a WDM-PAM4 OWC system using an 8-wavelength system as a demonstration; each wavelength carries a 50-Gb/s PAM4 signal data stream (50 Gb/s/λ × 8 λs). The scenario differences between this research and our previous research^16^ are illustrated in Table 1. Clearly, a UWLT system through the turbid water–air–turbid water link has a lot of room for improvement when considering the incorporation of FSO communication with the UWLT system. By utilizing a pair of doublet lenses in free-space transmission^17,18^, a laser beam reducer/expander and a reflective spatial light modulator (SLM) with an angle expander in water–air–water link^19–21^, good low bit error rate (BER) and acceptable PAM4 eye pattern are obtained over a 200-m free-space transmission with either an 8.8-m piped water–air–piped water link or a 6.5-m turbid water–air–turbid water link. Because the light at 1550 nm is less attenuated by the atmosphere than visible light^22,23^ and because an erbium-doped fiber amplifier (EDFA) can only magnify optical signals in the 1550 nm region, the wavelengths from 1535.82 to 1541.35 nm are selected in the FSO link of the OWC system. By using the low absorption of the piped water at the blue-light (B-light) wavelength^24,25^, a 450.6-nm B-light laser diode (LD) employing two-stage light injection technique is adopted through the piped water–air–piped water link. Moreover, by using the low attenuation of the turbid water at the red-light (R-light) wavelength^26,27^, a 660.3-nm R-light LD employing two-stage light injection technique is adopted through the turbid water–air–turbid water link.

**Table 1:** The scenario differences between this research and our previous research.

Research	Scenario			
	Architecture	Transmission	Transmitter	Tracking scheme
Our previous research	50 Gb/s PAM4 UWLT system	Water–air–water link	VCSEL with light injection and optoelectronic feedback techniques	Reflective SLM
This research	40 Gb/s WDM-PAM4 OWC system	Free-space transmission with water–air–water link	(1) Eight WDM wavelengths (2) R/B-light LD with two-stage light injection techniques	Reflective SLM with angle expander

One of the main challenges of the water–air–water link is the movement of the laser beam (lateral/vertical movement) produced by the water flow^28,29^. In actual situations, the movement of the laser beam due to water flow will cause the link to be unstable and lead to performance degradation. Therefore, laser beam tracking through the water–air–water interface is crucial in a UWLT system. A reflective SLM with an angle expander is thus used as a laser beam tracking scheme to reduce the movement of the laser beam caused by the water flow. An SLM with an angle expander not only operates as a flexible mirror to adaptively reflect the laser beam and maintain a reliable water–air–water link, but also solves the key problem currently being encountered by UWLT systems.

A WDM-PAM4 OWC system with an accumulative transmission rate of 400 Gb/s over 200-m free-space transmission with 8.8-m piped water–air–piped water link/6.5-m turbid water–air–turbid water link is successfully built. With SLM and angle expander-based beam tracking scheme, the laser beam can be controlled to mitigate its movement due to water flow, and the practical engineering problem can be solved to establish a stable OWC system. Compared with previous OWC systems through the free-space transmission with an air–water–air link^6–11^, it presents an excellent system with the advantages of high accumulative transmission rate, long-distance optical wireless transmission, and high reliability.

## Results

### The optical transmittances at different particle concentrations

**Figure 1:**
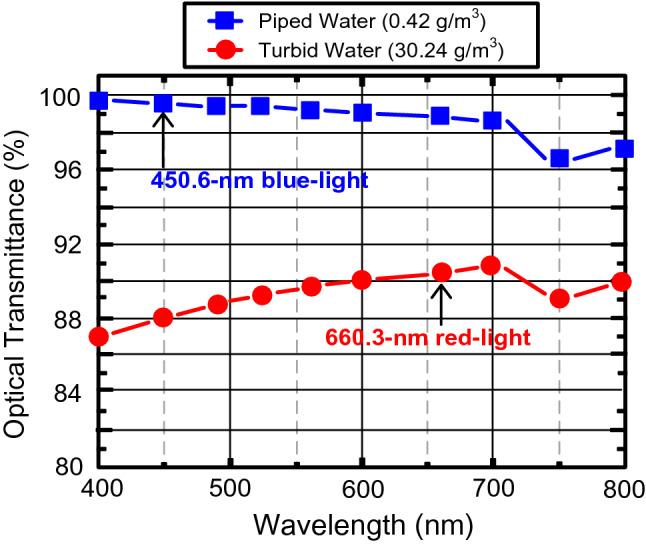
The optical transmittances at particle concentrations of 0.42 (piped water) and 30.24 g/m^3^ (turbid water).

The optical transmittances at particle concentrations of 0.42 (piped water) and 30.24 g/m^3^ (turbid water) are presented in Fig. 1. At a particle concentration of 0.42 g/m^3^ (piped water), the optical transmittance of B-light with a wavelength of 450.6 nm is higher than that of R-light with a wavelength of 660.3 nm. Thus, B-light outperforms R-light through the piped water–air–piped water interface. At a 30.24 g/m^3^ (turbid water) particle concentration, the optical transmittance of R-light with a wavelength of 660.3 nm is higher than that of B-light with a wavelength of 450.6 nm. Thus, R-light outperforms B-light through the turbid water–air–turbid water interface. In a piped water link, R-light suffers from greater absorption, and its advantage in terms of less scattering is not obvious. In a turbid water link, B-light suffers from greater scattering, and its advantage in terms of less absorption is not obvious. In conclusion, B-light is better for a piped water–air–piped water interface, whereas R-light is better for a turbid water–air–turbid water interface^30,31^.

### BER performances of 400 Gb/s WDM-PAM4 OWC system through 200 m free-space transmission and through 200 m free-space transmission with 8.8 m piped water–air–piped water link, and eye diagrams of 50 Gb/s PAM4 signal in different states

**Figure 2:**
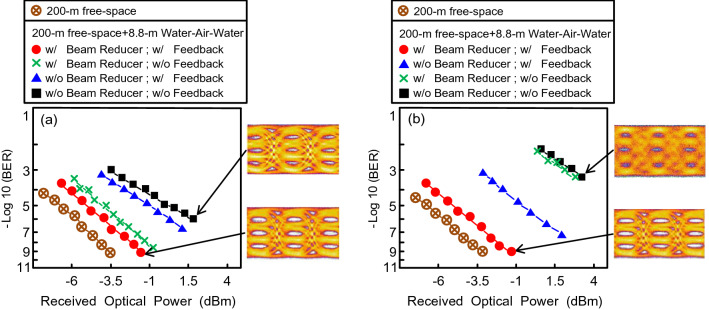
(**a**) BER performances of the 400 Gb/s WDM-PAM4 OWC system through 200 m free-space transmission and through 200 m free-space transmission with 8.8 m piped water–air–piped water link, at the beginning (within the first 5 min) and at a filtered wavelength of λ_1_. (**b**) BER performances of 400 Gb/s WDM-PAM4 OWC system under different scenarios, after an hour and at a filtered wavelength of λ_7_.

Figure 2a shows the BER performances of the 400 Gb/s WDM-PAM4 OWC system through 200 m free-space transmission and through 200 m free-space transmission with 8.8 m piped water–air–piped water link, at the beginning (within the first 5 min) and at a filtered wavelength of λ_1_. As BER reaches 10^−9^, a power penalty of approximately 2 dB exists between the condition through 200 m free-space transmission and that through 200 m free-space transmission with 8.8 m piped water–air–piped water link. This 2-dB power penalty is chiefly ascribed to the atmospheric attenuation from the 6.28 m free-space transmission and the absorption from the 2.52 m piped water link. Moreover, in the absence of a feedback signal (with laser beam reducer), BER increases slightly from 10^−9^ to 6 × 10^−9^. At the beginning, the movement of laser beam caused by the water flow is very small. Therefore, in the absence of a feedback signal, BER increases slightly. Furthermore, in the absence of a laser beam reducer (with feedback signal), BER increases to 3 × 10^−7^. In the absence of a laser beam reducer and feedback signal, BER further increases to 10^−6^. Using a laser beam reducer, BER reaches an order of 10^−9^. Without using a laser beam reducer, BER increases to an order of 10^−7^ ~ 10^−6^. At the beginning, a laser beam reducer is an important factor in the UWLT system through the piped water–air–piped water link. Regarding PAM4 eye diagrams, open eye diagrams are observed in the presence of a laser beam reducer and feedback signal. In the absence of a laser beam reducer and feedback signal, a more or less clear eye diagram is obtained.

Figure 2b displays the BER performances of the 400 Gb/s WDM-PAM4 OWC system under different scenarios, after an hour and at a filtered wavelength of λ_7_. At a value of 10^−9^ BER, a power penalty of about 2 dB occurs between the scenario through 200 m free-space transmission and that through 200 m free-space transmission with 8.8 m piped water–air–piped water link. The 2-dB power penalty results from the atmospheric attenuation because of the 6.28 m free-space transmission and the absorption because of the 2.52 m piped water link. Furthermore, in the absence of a laser beam reducer (with feedback signal), BER increases to 5.2 × 10^−8^. Moreover, in the absence of a feedback signal (with or without laser beam reducer), BER significantly increases to 4.3 × 10^−4^. Over time (after an hour), the movement of laser beam produced by the water flow is quite large. However, the feedback signal supplied in an electrical controller can arbitrarily adapt the laser beam and thereby mitigate the movement of laser beam. Over time, a feedback signal is the key factor in the UWLT system through the piped water–air–piped water link. Figure 2b also shows the eye diagrams of the 50 Gb/s PAM4 signal in different states. In the presence of a laser beam reducer and feedback signal, open eye diagrams are attained. In the absence of a laser beam reducer and feedback signal, blurred eye diagrams are acquired.

### BER performances of 400 Gb/s WDM-PAM4 OWC system through 200 m free-space transmission and through 200 m free-space transmission with 6.5 m turbid water–air–turbid water link, and eye diagrams of 50 Gb/s PAM4 signal in different conditions

**Figure 3:**
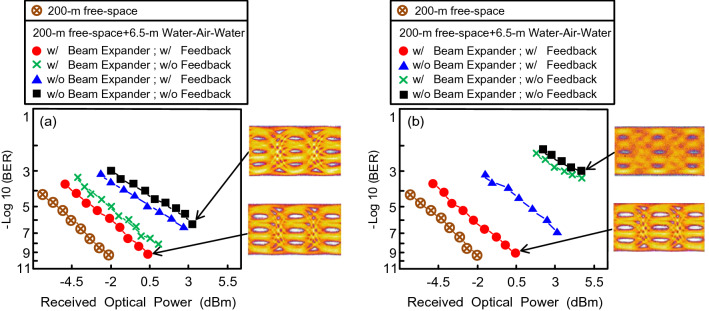
(**a**) At the beginning (within the first 5 min) and at a filtered wavelength of λ_2_, the BER performances of the 400 Gb/s WDM-PAM4 OWC system through 200 m free-space transmission and through 200 m free-space transmission with 6.5 m turbid water–air–turbid water link. (**b**) After an hour and at a filtered wavelength of λ_8_, the BER performances of the 400 Gb/s WDM-PAM4 OWC system under different conditions.

At the beginning (within the first 5 min) and at a filtered wavelength of λ_2_, the BER performances of the 400 Gb/s WDM-PAM4 OWC system through 200 m free-space transmission and through 200 m free-space transmission with 6.5 m turbid water–air–turbid water link is shown in Fig. 3a. At a 10^−9^ BER value, a power penalty of around 2.5 dB exists between the state over 200 m free-space transmission and that over 200 m free-space transmission with 6.5 m turbid water–air–turbid water link. Because scattering is the dominant factor in a turbid water link, this 2.5-dB power penalty mainly arises from the atmospheric attenuation due to 5 m free-space transmission and the scattering effect due to 1.5 m turbid water link. In addition, in the absence of a feedback signal (with laser beam expander), BER somewhat increases from 10^−9^ to 9.2 × 10^−9^. At the beginning, the movement of laser beam induced by the water flow is very small. The feedback signal thereby affords a slight improvement in BER performance. Moreover, in the absence of a laser beam expander (with feedback signal), BER increases to 6.5 × 10^−7^. In the absence of a laser beam expander and feedback signal, BER further increases to 3 × 10^−6^. At the beginning, the laser beam expander is the crucial factor in the UWLT system through the turbid water–air–turbid water link. Concerning PAM4 eye diagrams, open eye diagrams are acquired in the condition with a laser beam expander and feedback signal. In the condition without a laser beam expander and feedback signal, a somewhat clear eye pattern is attained.

The BER performances of the 400 Gb/s WDM-PAM4 OWC system after an hour and at a filtered wavelength of λ_8_ under different conditions are presented in Fig. 3b. As BER is 10^−9^, a 2.5-dB power penalty emerges between the condition over 200 m free-space transmission and that over 200 m free-space transmission with 6.5 m turbid water–air–turbid water link. This 2.5-dB power penalty is mostly attributed to the atmospheric attenuation from the 5 m free-space transmission and the scattering effect from the 1.5 m turbid water link. Moreover, in the condition with a feedback signal but without a laser beam expander, BER performance worsens to 8.5 × 10^−8^. In addition, in the condition without a feedback signal (with or without a laser beam expander), BER performance critically worsens to 8 × 10^−4^. Over time, the feedback signal is the influential factor in the UWLT system through the turbid water–air–turbid water link. Figure 3b also exhibits the 50 Gb/s PAM4 signal’s eye diagrams under different conditions. In the condition with a laser beam expander and feedback signal, open eye diagrams are acquired. Whereas in the condition without a laser beam expander and feedback signal, the eye diagrams are almost closed.

## Discussion

For a UWLT system passing through the piped water–air–piped water link, the main factor is absorption, and the low scattering coefficient prevents the laser beam from diverging. Using a laser beam reducer to decrease the beam size will improve the performance of the UWLT system passing through the piped water–air–piped water link because the pipe water absorbs less laser light^32^. As for the UWLT system passing through the turbid water–air–turbid water interface, the avalanche photodiode (APD) with a trans-impedance amplifier (TIA) receiver will receive a large amount of scattered light when using a laser beam expander to increase the beam size^33,34^. Employing a laser beam expander to expand the beam size, the performance of the UWLT system through the turbid water–air–turbid water interface is improved. Since that the beam divergence is inversely proportional to the beam size, a reduced beam divergence occurs with a laser beam expander. A larger beam size that follows a smaller beam divergence contributes more scattered light received by the APD with a TIA receiver, and thus results in better performance. Nevertheless, a larger beam size accompanies a larger absorption. For a UWLT system passing through the turbid water–air–turbid water interface, given that the ratio of absorbed light is small, a smaller beam divergence with a larger beam size brings on a smaller amount of light absorbed by the turbid water. Consequently, the advantage of large beam size is attained. A laser beam reducer/expander is an important factor in improving the performance of the UWLT system through the piped/turbid water–air–piped/turbid water link.

**Figure 4:**
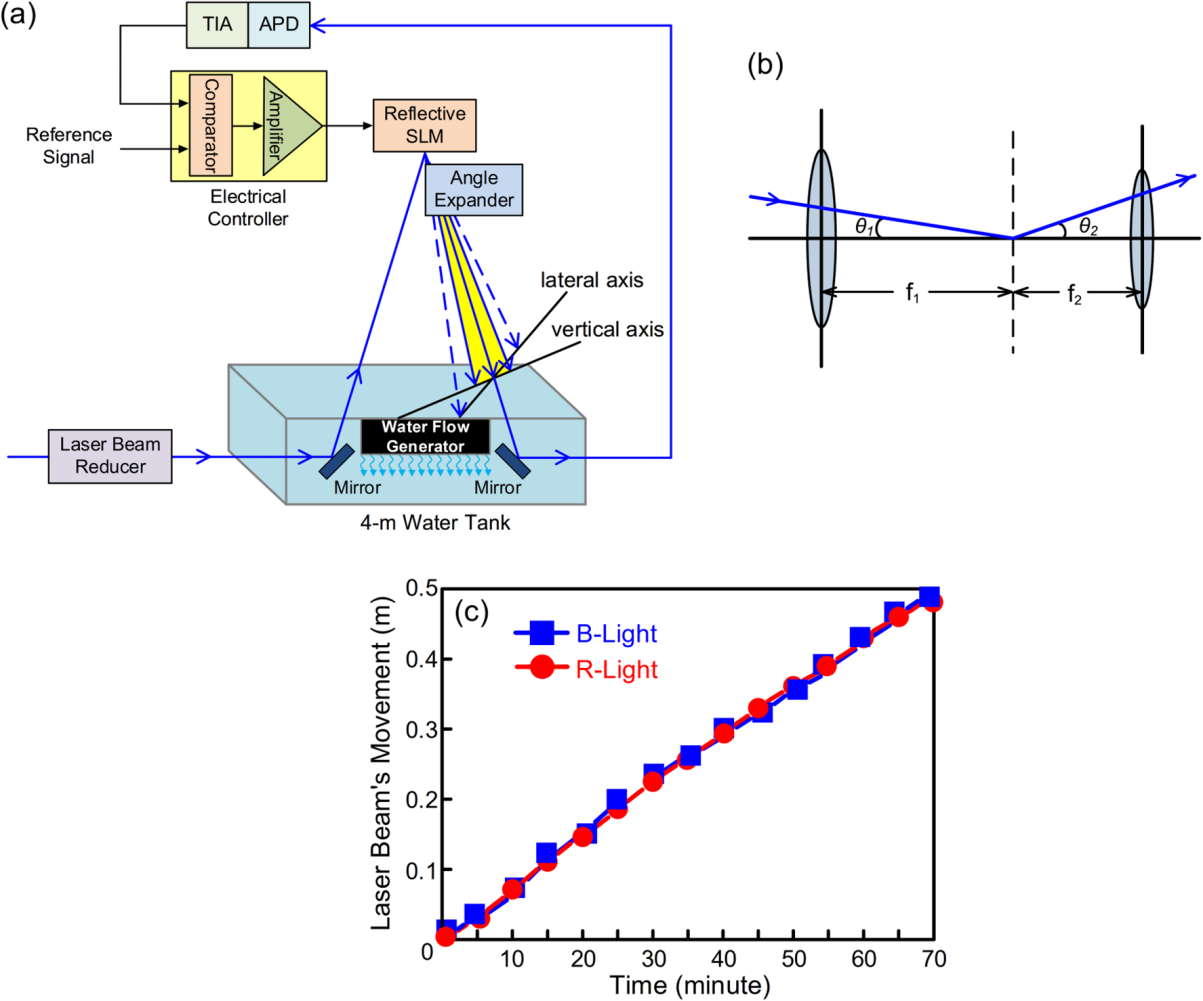
(**a**) The reflective SLM with an angle expander for developing a UWLT system across the water–air–water interface and mitigating link interruption caused by the movement of the laser beam (lateral or vertical movement). (**b**) The angle expander based on an afocal system, which comprises two convex lenses with *f*_1_ (50 mm) and *f*_2_ (25.4 mm) focal lengths. (**c**) The movement of the laser beam (lateral movement) over 70 min.

The reflective SLM with an angle expander for developing a UWLT system across the water–air–water interface and mitigating link instability caused by the movement of the laser beam (lateral or vertical movement) is shown in Fig. 4a. Water flow brings on the movement of the laser beam, causing the APD with a TIA receiver to receive less light, thereby generating a low-level signal. The electrical controller receives the reference signal and the low-level signal, compares them, generates an amplified output voltage to make the liquid crystal inside the SLM work, and adaptively adjusts the laser beam. Figure 4b illustrates the angle expander based on an afocal system, which comprises two convex lenses with *f*_1_ (50 mm) and *f*_2_ (25.4 mm) focal lengths^35^. The distance of two convex lenses is equal to the sum of the focal lengths (*f*_1_ + *f*_2_). The function of the angle expander is to magnify the output beam angle (*θ*_2_):

1 $$\theta_{2} = \theta_{1} \cdot \left( {\frac{{f_{1} }}{{f_{2} }}} \right)$$

where *θ*_1_ is the input beam angle, and *f*_1_/*f*_2_ (50 mm/25.4 mm ~ 2) is the focal length ratio (magnification factor). Clearly, output beam angle increases with the increase in focal length ratio. After passing through an angle expander, a reflective SLM with ± 5° diffraction angle is expanded into a ± 10° diffraction angle. Within a beam tracking angle of ± 10°, the laser beam can have a larger lateral/vertical beam tracking range to make up for the BER decline produced by the movement of the laser beam. Given that the reflective SLM with an angle expander has a diffraction angle of ± 10°, the lateral/vertical beam tracking range (*TR*) in the UWLT system through the piped water–air–piped water interface can be extended from ± 0.29 m to ± 0.58 m:

2 $$TR = 3.4 \times \left( {5 \times \frac{\uppi }{180}} \right) = 0.29\;({\text{m}})$$

3 $$TR = 3.4 \times \left( {10 \times \frac{\uppi }{180}} \right) = 0.58\;({\text{m}}).$$

Meanwhile, the lateral/vertical beam tracking range in the UWLT system through the turbid water–air–turbid water link can be extended from ± 0.24 m to ± 0.48 m:

4 $$TR = 2.75 \times \left( {5 \times \frac{\uppi }{180}} \right) = 0.24\;({\text{m}})$$

5 $$TR = 2.75 \times \left( {10 \times \frac{\uppi }{180}} \right) = 0.48\;({\text{m}}).$$

The values of ± 0.58 m and ± 0.48 m, taken from Eqs. (3) and (5), indicate the maximum lateral/vertical beam tracking ranges. In the lateral/vertical beam tracking range, the laser beam can be arbitrarily adapted to mitigate the BER deterioration caused by the flow of water.

The movement of the laser beam (lateral movement) over 70 min is presented in Fig. 4c. Obviously, the movement of laser beam increases with the increase in time. In the first 5 min, the movement of laser beam caused by the water flow is very small. After an hour, nevertheless, the movement of laser beam produced by the water flow is quite large. Although the average speed of the water flow in the first 5 min and after an hour is nearly the same (approximately 1.5 m/s), however, the movement of laser beam due to water flow will accumulate as time increases. For example, in the first 5 min, the movement of laser beam caused by the water flow is 0.03 m. In the first 10 min, the movement of laser beam produced by the water flow moves from 0.03 to 0.07 m, instead of from 0 to 0.04 m. Therefore, the longer the time, the larger the movement of the laser beam.

## Methods

### Architecture of the 400 Gb/s WDM-PAM4 OWC system through a 200-m free-space transmission with either an 8.8-m piped water–air–piped water link or a 6.5-m turbid water–air–turbid water link

**Figure 5:**
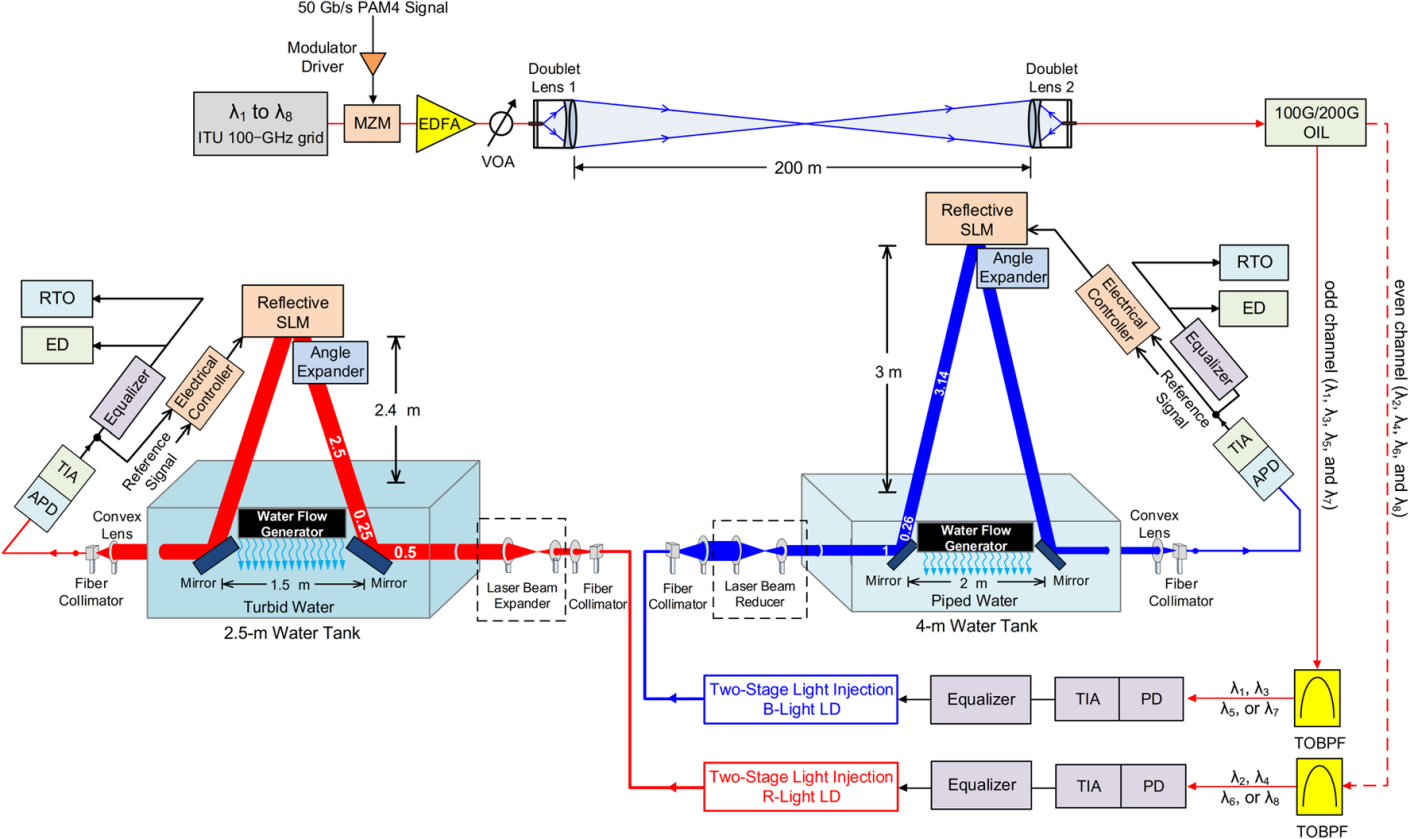
The architecture of the 400 Gb/s WDM-PAM4 OWC system through 200 m free-space transmission with either 8.8 m piped water–air–piped water link or 6.5 m turbid water–air–turbid water link.

**Figure 6:**
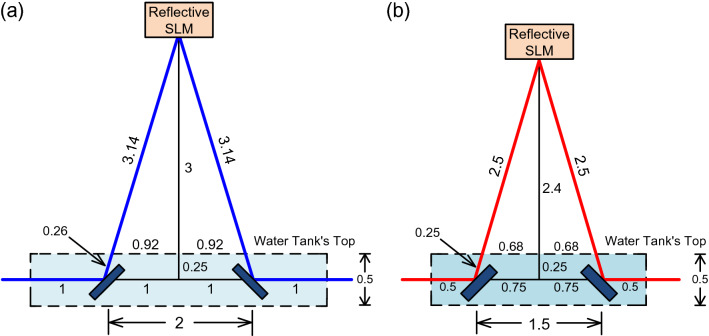
(**a**) An 8.8-m (1 × 2 + 0.26 × 2 + 3.14 × 2) piped water–air–piped water link. (**b**) A 6.5-m (0.5 × 2 + 0.25 × 2 + 2.5 × 2) turbid water–air–turbid water link.

The architecture of the 400 Gb/s WDM-PAM4 OWC system through a 200-m free-space transmission with either an 8.8-m piped water–air–piped water link or a 6.5-m turbid water–air–turbid water link is presented in Fig. 5. Eight WDM wavelengths from 1535.82 to 1541.35 nm with 100 GHz spacing are used as optical carriers. These eight optical carriers from λ_1_ to λ_8_ are fed into a Mach–Zehnder modulator that is modulated by a 50-Gb/s PAM4 signal generated from the PAM4 signal generator. Then, the optical signals are amplified by an EDFA with an output power of 16 dBm at an input power of 0 dBm, optimally reduced by a variable optical attenuator, and transmitted by a couple of doublet lens over 200 m free-space transmission. The transmitted optical signals are supplied in a 100 GHz/200 GHz optical inter-leaver (OIL) to separate odd (even) wavelengths. The outputs of the OIL are sent to different tunable optical band-pass filters (TOBPFs) with 0.44 nm 3-dB bandwidth and 500 dB/nm filter slope to filter the desired optical wavelengths. The demultiplexing scheme is composed of one OIL and two TOBPFs. The function of the demultiplexing scheme is to distinguish each optical wavelength from the output of the demultiplexing scheme. Next, a 22-GHz PD with a TIA receiver, with 4.5 V PD reverse bias voltage and 0.91 A/W responsivity at 1540 nm, receives and enhances the filtered optical signal. If eight optical wavelengths are distinguished at the same time, then two 1 × 4 WDM demultiplexers (or one 1 × 8 WDM demultiplexer) and eight PDs with TIA receivers are required to separate, receive, and enhance each optical wavelength. However, there will be crosstalk that arises from the incomplete isolation of the adjacent channels. Such crosstalk will cause performance degradation. Moreover, these eight PDs with TIA receivers will increase the cost of WDM-PAM4 OWC systems. For a real implementation of WDM-PAM4 OWC system, it is necessary to develop a low-cost demultiplexing scheme. An equalizer is used to equalize the boosted 50 Gb/s PAM4 signal. After equalization, the 50 Gb/s PAM4 signal is fed into a 450.6-nm B-light/660.3-nm R-light LD based on a two-stage light injection technique^36,37^. The 450.6-nm B-light LD has an optical output power of 1 dBm (30 mA operating current) to 6 dBm (42 mA operating current), and the 660.3-nm R-light LD has an optical output power of 3.5 dBm (42 mA operating current) to 8 dBm (55 mA operating current). The laser beam emitted from the 450.6-nm B-light LD employing two-stage light injection technique is inputted into a laser beam reducer to reduce the laser beam’s diameter from 2.2 mm to 1.1 mm. The reduced laser beam is transmitted through a water tank with a size of 4 m × 0.5 m × 0.5 m (length × width × height). The water tank is filled with piped water with a particle concentration of 0.42 g/m^3^. Two plane mirrors, with a separation distance of 2 m, are placed in the water tank to reflect the laser beam through the piped water–air–piped water link. A reflective SLM, with 5 ms response time, 95% reflectivity and ± 5° diffraction angle, is placed above the water tank to reflect the downstream laser beam adaptively. Response time is the key parameter of reflective SLM. Because the average speed of the water flow is about 1.5 m/sec, the response time (5 ms) of the reflective SLM can satisfy the requirement to adaptively adjust the laser beam passing through the water–air–water interface. To achieve a high reflectivity of 95%, a mirror coating is put on the backplane of the reflective SLM. Subsequently, an angle expander is employed to expand the reflection angle of the downstream laser beam. Given that the distance between the reflective SLM and the top of the water tank is 3 m, an 8.8-m (1 × 2 + 0.26 × 2 + 3.14 × 2) piped water–air–piped water link, as shown in Fig. 6a, is attained. A water flow generator with a speed of 1.5 m/sec is utilized to simulate water flow. After reflection by the right-side plane mirror in the water tank, the laser beam is concentrated by a convex lens to guide it into a fiber collimator, and then amplified by a 25-GHz avalanche PD (APD) with a TIA receiver with 20 V APD reverse bias voltage and 32 A/W responsivity at 450.6 nm. A part of the amplified 50 Gb/s PAM4 signal (feedback signal) is supplied in an electrical controller to adapt the reflected laser beam. Another part of the amplified 50 Gb/s PAM4 signal is supplied in an equalizer with a 28-Gb/s error detector (ED) to evaluate BER performances in real-time. Furthermore, a real-time oscilloscope (RTO) is utilized to seize the eye diagrams of the 50 Gb/s PAM4 signal.

The laser beam emitted from the 660.3 nm R-light LD employing two-stage light injection technique is supplied in a laser beam expander to increase the laser beam’s diameter from 2.2 mm to 4.4 mm. The expanded laser beam is delivered across a water tank with a dimension of 2.5 m × 0.5 m × 0.5 m (length × width × height). The water tank is filled with turbid water with a particle concentration of 30.24 g/m^3^. Two plane mirrors, with a separation distance of 1.5 m, are placed in the water tank to reflect the laser beam across the turbid water–air–turbid water interface. A reflective SLM with an angle expander is placed above the water tank to adjust the downstream laser beam adaptively. Because the distance between the reflective SLM and the water tank’s top is 2.4 m, a 6.5-m turbid water–air–turbid water link, as presented in Fig. 6b, is obtained. A water flow generator with a speed of 1.5 m/sec is utilized to simulate water flow. After reflection by the left-side plane mirror in the water tank, the laser beam is focused by a convex lens to conduct it into a fiber collimator, then improved by a high-bandwidth APD with a TIA receiver with 20 V APD reverse bias voltage and 25 A/W responsivity at 660.3 nm. A portion of the improved 50 Gb/s PAM4 signal (feedback signal) is fed into an electrical controller to attune the reflected laser beam adaptively. Another portion of the enhanced 50 Gb/s PAM4 signal is fed into an equalizer with a high-sensitivity ED for real-time BER measurement. Additionally, an RTO is used to take the 50 Gb/s PAM4 signal’s eye diagrams.
